# 

**Published:** 1905-09

**Authors:** 


*The following donations have been received since the publication of the List in June:*

*August 31st, 1905.*

| | |
|---|---|
| J. S. Ashmead (1) ... ... ... ... ... ... ... ... | 1 volume. |
| Medical Officers of Health of Bristol Port Sanitary District (2) ... ... ... ... ... ... ... ... | 1 ,, |
| Nelson C. Dobson (3) ... ... ... ... ... ... ... ... | 26 volumes. |
| L. M. Griffiths (4) ... ... ... ... ... ... ... ... | 4 ,, |
| The Editor of *Virginia Medical Semi-Monthly* (5) ... | 1 volume. |

Unbound periodicals have been received from Dr. R. Shingleton Smith, and unframed portraits from Mr. J. S. Ashmead.

### FIFTY-SEVENTH LIST OF BOOKS.

The titles of books mentioned in previous lists are not repeated.

The figures in brackets refer to the figures after the names of the donors, and show by whom the volumes were presented. The books to which no such figures are attached have either been bought from the Library Fund or received through the *Journal*.

| | | | |
|---|---|---|---|
| **Acland, T. D.** ... | *On the Hours of Sleep at Public Schools* ... ... ... | | 1905 |
| **Bidwell, L. A.** ... | *A Handbook of Intestinal Surgery* ... ... ... ... ... | | 1905 |
| **Braidwood, P. M.** | *Pyæmia* ... ... ... ... ... ... ... ... ... ... | (3) | 1868 |
| **Browne, J. H. B.** | *Medical Jurisprudence of Insanity* ... ... ... ... | (3) | 1871 |
| **Chambers, G. F.** . | *Public Health* ... ... ... ... ... ... ... | (3) 8th Ed. | 1881 |
| **Clark, F. Le G.** ... | *Outlines of Surgery* ... ... ... ... ... ... | (3) 2nd Ed. | 1872 |
| ,, | *Papers on Surgery, Pathology, &c.* ... ... ... | (3) | 1889 |
| **Clarke, J. M.** ... | *Hysteria and Neurasthenia* ... ... ... ... ... ... ... | | 1905 |
| **Cohn, T.** ... ... | *Methodische Palpation.* Teil I. ... ... ... ... ... ... | | 1905 |
| **Cohnheim, P.** ... | *Die Krankheiten des Verdauungskanal* ... ... ... ... | | 1905 |
| **Cripps, W. H.** ... | *Cancer of the Rectum* ... ... ... ... ... ... ... | (3) | 1880 |
| **Curgenven, J. S.** . | *The Child's Diet* ... ... ... ... ... ... ... ... | | 1905 |
| **Duhring, L. A.** ... | *Atlas of Skin Diseases* ... ... ... ... ... ... ... | (3) | 1876 |
| **Fox, T.** ... ... | *Atlas of Skin Diseases* ... ... ... ... ... ... ... | (3) | 1877 |
| **Freeland, E. H.** .. | *The Radical Cure of Corns and Bunions* ... | 2nd Ed. | 1905 |
| **Gerster, A. G.** ... | *Aseptic and Antiseptic Surgery* ... ... ... ... ... | (3) | 1888 |
| **Heath, C.** ... ... | *Course of Operative Surgery* ... ... ... ... ... ... | (3) | 1877 |
| **Herschell, G.** ... | *Electrical Methods in Affections of the Stomach* ... ... | | 1905 |
| **Humphry, G. M.** . | *Old Age* ... ... ... ... ... ... ... ... ... ... ... | (3) | 1889 |
| **Huxley, T. H.** ... | *Anatomy of Invertebrate Animals* ... ... ... ... | (3) | 1877 |
| **Ledwich, E. L'E.** | *Anatomy of the Inguinal and Femoral Regions* ... | (3) | 1884 |
| **Lowe, T. P.** ... | *Bath Thermal Waters* ... ... ... ... ... ... ... ... | (4) | 1890 |
| **Lowne, B. F.** ... | *Ophthalmic Surgery* ... ... ... ... ... ... ... ... | (3) | 1876 |
| **Macgregor, Jessie M.** | *Pathology of the Endometrium* ... ... ... ... ... ... | | 1905 |
| **Moinet, F. W.** ... | *Medical Examination for Life Assurance* ... ... | (3) | 1876 |
| *Pamphlets* ... ... | 13 vols. ... ... ... ... ... ... ... ... ... ... ... ... | | [V.Y.] |
| **Proctor, G. B.** ... | *Notes on How to Rear an Infant* ... ... ... ... ... | | [N.D.] |
| **Radcliffe, C. B.** ... | *Vital Motion as a Mode of Physical Motion* ... ... | (3) | 1876 |
| **Ranney, A. L.** ... | *Surgical Diagnosis* ... ... ... ... ... ... | (3) 2nd Ed. | 1881 |
| **Reed, V. C. Vaughan and E. O. Shakespeare, W.** | *Report on Typhoid Fever in U.S. Camps in Spanish War of 1898.* 2 vols. | | 1904 |
| **Sajous, C. E.** ... | *Diseases of the Nose and Throat* ... ... ... ... ... | (3) | 1889 |
| **Shakespeare, W. Reed, V. C. Vaughan and E. O.** | *Report on Typhoid Fever in U.S. Camps in Spanish War of 1898.* 2 vols. | | 1904 |
| **Simpson, Eve B.** . | *Dogs of Other Days* ... ... ... ... ... ... ... ... | (1) | 1882 |
| **Stein, A. W.** ... | *Tumors of the Bladder* ... ... ... ... ... ... ... | (3) | 1881 |
| **Sutherland, W. G.** | *Dispensing made Easy* ... ... ... ... ... ... | 2nd Ed. | 1905 |
| **Symes, A. D. Waller and W. L.** | *Exercises in Practical Physiology.* Part II. | | 1905 |
| **Tait, L.** ... ... | *Diseases of Women and Abdominal Surgery.* Vol. I. | (3) | 1889 |
| **Thompson, J. A.** . | *Free Phosphorus in Medicine* ... ... ... ... ... ... | (3) | 1874 |
| **Turnbull, L.** ... | *Diseases of the Ear* ... ... ... ... ... ... ... ... | (3) | 1872 |

**Vaughan and E. O. Shakespeare, W. Reed, V. C.** *Report on Typhoid Fever in U.S. Camps in Spanish War of 1898.* 2 vols. . 1904
**Wagstaffe, W. W.** *Human Osteology* ... ... ... ... ... ... ... (3) 1875
**Waller and W. L. Symes, A. D.** *Exercises in Practical Physiology.* Part II. 1905
**Watson, J. R.** ... *Natural Science in Hygiene* ... ... ... ... ... ... ... 1905
**Wells, Sir T. S.** ... *Ovarian and Uterine Tumours*... ... ... ... ... (3) 1882
**White, F. F.** ... *The Rational Treatment of Running Ears* ... ... ... 1905
**Ziemssen, H. von** *Handbook of Skin Diseases* ... ... ... ... ... (3) 1885

### TRANSACTIONS, REPORTS, JOURNALS, &c.

American Journal of Ophthalmology, The ... ... ... Vol. XXI. 1904
American Journal of the Medical Sciences, The Vols. CXXVIII., CXXIX. 1904-05
American Medicine ... ... ... ... ... ... ... ... ... Vol. IX. 1905
American Pediatric Society, Transactions of the ... ... Vol. XVI. 1904
Archives provinciales de Chirurgie ... ... ... ... ... Tom XIII. 1904
Archivio di Ortopedia ... ... ... ... ... ... ... ... ... ... ... 1904
Bristol Medico-Chirurgical Journal, The ... ... ... ... Vol. XXII. 1904
Bristol Port Sanitary District, Annual Report for 1904 ... ... ... (2) 1905
British Gynæcological Journal, The ... ... ... ... ... Vol. XX. 1904-05
British Medical Journal, The ... ... ... ... ... ... Vol. I. for 1905
Bulletin de l'Académie royale de Medécine de Belgique ... Tom. XVIII. 1904
Cincinnati Lancet-Clinic, The ... ... ... ... ... Vol. LXXXIII. 1905
Clinical Journal, The ... ... ... ... ... ... ... ... Vol. XXV. 1905
Edinburgh Medical Journal, The ... ... ... ... N.S., Vol. XVII. 1905
Hospital, The ... ... ... ... ... ... ... ... ... ... Vol. XXXVII. 1905
Hospitals—
Westminster Hospital Reports, The ... ... ... ... Vol. XIV. 1905
Johns Hopkins Hospital Bulletin, The ... ... ... ... Vol. XV. 1904
Journal of Experimental Medicine, The ... ... ... ... Vol. VI. 1901-05
Journal of Mental Pathology, The ... ... ... ... Vols. V., VI. 1903-04
Journal of Obstetrics and Gynæcology of the British Empire, The Vol. VII. 1905
Journal of Tropical Medicine, The ... ... ... ... ... Vol. VII. 1904
Journal of the American Medical Association, The ... Vol. XLIII. 1904
Journal of the Royal Sanitary Institute ... ... ... ... Vol. XXV. 1904
Lancet, The ... ... ... ... ... ... ... ... ... ... ... Vol. I. for 1905
Lister Institute of Preventive Medicine, The. Collected Papers. No. I. 1904
Louisville Monthly Journal of Medicine and Surgery, The Vol. XI. 1904-05
Medical Chronicle, The ... ... ... ... .. 4th Ser., Vol. VIII. 1904-05
Medical News, The ... ... ... ... ... Vols. LXXXV., LXXXVI. 1904-05
Medical Press and Circular, The ... ... ... ... [Vol. CXXX.] 1905
Medical Record ... ... ... ... ... ... ... ... ... ... ... Vol. LXVII. 1905
Münchener medizinische Wochenschrift ... ... ... ... Bd. I. for 1905
Obstetrical Transactions ... ... ... ... ... ... ... ... Vol. XLVI. 1905
Philadelphia County Medical Society, Proceedings of the Vol. XXV. 1904
Practical Medicine ... ... .. ... ... ... ... ... ... ... Vol. II. 1904
Progressive Medicine .. ... ... ... ... ... ... ... ... Vol. II. for 1905
St. Petersburger medicinische Wochenschrift ... ... ... ... ... ... 1904

| | | |
|---|---|---|
| South African Medical Record, The | Vol. II. | 1904 |
| Treatment | Vol. VIII. | 1904-05 |
| University of Pennsylvania Medical Bulletin | Vol. XVII. | 1905 |
| Virginia Medical Semi-Monthly | (5) Vol. IX. | 1905 |
| West London Medical Journal | Vol. IX. | 1904 |

### Obituary Notices.

### Local Medical Notes.

**University College, Bristol.**—The following successes by Students have recently been recorded:—

UNIVERSITY OF LONDON.—*M.B. Intermediate Examination:* Cecil Clarke, L. J. Short ("Distinguished in Pharmacology," Scholarship in Pharmacology), J. M. Hammond.

CONJOINT BOARD OF ENGLAND. — *Chemistry and Physics:* C. H. Hart, H. R. B. Hull, W. A. Reynolds, T. S. Rippon. *Elementary Biology:* G. H. Griffiths, H. R. B. Hull, J. F. H. Morgan, A. J. O. Wigmore. *Practical Pharmacy:* P. Sinnock. *Medicine:* R. O. Bodman, W. J. H. Pinniger,* J. B. V. Watts, M. R. O. Wilson. *Surgery:* F. G. Bergin, W. J. H. Pinniger,*

Lionel Shingleton Smith,* A. J. M. Wright. *Midwifery:* V. B. Green-Armytage, P. Moxey, G. S. Parkinson, H. K. Salisbury.

L.D.S (ENG.)—*Chemistry and Physics:* I. R. Huddleston. *Mechanical Dentistry:* F. F. Hatton, F. C. Nicholls. *Dental Metallurgy:* C. A. Joll, F. C. Nicholls.

L.S.A.—*Surgery:* A. H. Hughes.*

**Bristol Royal Infirmary.**—J. J. S. Lucas, M.D. Lond., B.A., has been appointed Honorary Pathologist. Junior House Surgeon, A. L. Sheppard. Casualty Officer, L. Shingleton Smith.

**Bristol General Hospital.**—H. A. Knight, M.B., B.Ch. Edin., D.P.H., has been appointed Assistant House-Physician.

**North Eastern Hospital, London.** — Theodore Fisher, M.D. Lond., M.R.C.P., has been appointed Pathologist to this hospital.

**Clifton College.**—Dr. J. O. Symes has been appointed Physician to the college.

**County Hospital, Newport, Mon.**—Mr. V. A. Crinks, M.R.C.S., L.R.C.P., has been appointed House-Surgeon.

**Winsley Sanatorium.**—E. Dunbar Townroe, M.D., has been appointed Resident Medical Officer.

* Completes Examination.

### BRISTOL.

**Port Sanitary District.**—The annual report of the Medical Officer of Health states that 346 ships came from infected ports and they were all examined. No case of plague, cholera, or small-pox was found, but a few of enteric fever were dealt with. A river ambulance service has been established, which is proving useful. About two dozen cases were admitted to the hospital ship *Margarida.*

**Royal Infirmary.**—A large carnival has recently been held in the Zoological Gardens in aid of the Infirmary. Sir George White, Bart., the President, has been the leading spirit in the inception and execution of this idea for helping the Infirmary, and was supported by a large number of willing helpers. The Duchess of Beaufort became deeply interested in the scheme and induced a large number of ladies to take an active part in carrying it out, and herself declared the carnival open. Bristol thoroughly gave itself over to the spirit of festivity with a laudable object which cleared the Infirmary of the debt of £15,552 18s. 2d., of which sum more than half was contributed by Sir George White, Bart., and the large sum of £4,015 18s. 11d. was contributed by Mr. Samuel White towards the expenses.

### BATH.

**Winsley Sanatorium.**—The Bath and Bristol Branch of the British Medical Association was invited on July 12th to the Winsley Sanatorium, and Dr. Lionel Weatherly gave some interesting details of the institution since its opening last

December. During the period indicated 57 of the 60 beds have been occupied. Altogether 85 patients have been treated and 31 have been discharged. Of the latter number 7 have returned to their ordinary employment and 11 have found lighter work than they had previously been accustomed to. Eight patients were sent home without any signs of improvement, 9 were very much improved, and in 14 instances the arrest of the disease was practically complete. Dr. Weatherly spoke of the difficulty there was in finding suitable work with proper surroundings for those who had been treated in the Sanatorium, and suggested the formation of an after-care association for the purpose of dealing with such cases.

### CARDIFF.

**Cardiff Infirmary.**—The report of this institution for 1904 records an expenditure of £13,131 and an income of £11,477. There was, on an average, 156 patients in hospital daily, and the average cost per occupied bed was £65, or about 8s. less than in the previous year. Among the 2,144 patients admitted during the year 1,164 were treated in the surgical wards, 303 in the medical, 428 in the ophthalmic, and 249 in the gynæcological. With regard to the payment for medicines and dressings by out-patients, the Chairman of the Board of Management is anxious that some charge should be made, and Mr. Lynn Thomas has said that people came in from the outlying districts for 2s. worth of medicine and paid 2s. 9d. in railway fares, while they were quite well able to pay a local practitioner.

**The Royal Hamadryad Seamen's Hospital.**—This hospital was erected to succeed the old *Hamadryad* ship, which receives the sick and injured sailors who land at Cardiff, to commemorate the Diamond Jubilee of the late Queen Victoria, and the late Marquess of Bute associated himself with the project in the heartiest manner. There are three main wards, each containing 16 beds, and smaller wards, bringing up the total accommodation in the hospital to 60 beds. The foundation-stone was laid in 1902 by the Marquess of Bute, when he came of age, and was formerly opened by him on June 30th last.

### GLOUCESTER.

**Schools and Infection.**—A short circular bearing the signature of Dr. J. Middleton Martin, the Medical Officer to the Gloucester Education Committee, has recently been issued to the school teachers of the county dealing with the question of infection among school children. The teachers are advised, when there is reason to suspect that a child is suffering from an infectious disease, to send him home at once with a written note to the parent requesting that the child should be seen by a medical practitioner and a report of the opinion so obtained sent to the teacher. For this opinion it appears that the Education Committee is prepared to pay a fee of one shilling. In connection with the school closure, Dr. Martin points out that such a step taken in panic may do more harm than good, because a valuable source of information as to suspected mild unrecognised cases is thus thrown away. Accompanying the circular is a leaflet giving some details with regard to principal symptoms of infectious diseases and of contagious conditions which are liable to affect children. Included in the latter are scabies, ringworm, ophthalmia, impetigo contagiosa, and pediculosis.

**Health Report.**—In his annual report for 1904, the former Medical Officer of Health of the City of Gloucester (Dr. John Campbell) estimated the population of the city as 50,510, and recorded a death rate of 14.8 per 1,000, and an infantile mortality rate of 134 per 1,000 births. The death rate for pulmonary tuberculosis in 1904 was 0.9 per 1,000. Dr. Campbell advocates the voluntary notification of cases of this disease, and although he suggests the erection of wooden huts on the isolation hospital's estate for the treatment of suitable cases, he expresses the very distinct opinion that the prevention and cure of consumption are to be found in the creation of healthy homes and healthy surroundings for the people.

### PENZANCE.

**The West Cornwall Infirmary.**—This new institution at Penzance is now nearly completed, and it is hoped that it will be ready for the reception of patients in about three months. The cost of the building will be about £8,000. The hospital comprises a central administrative block of two storeys, having a ward on each side. From the south wing of the building the new public dispensary will be built. A well-fitted operating theatre and a laundry, &c., have been added.

### SWANSEA.

**General Hospital.**—In the last annual report of this institution it is stated that the hospital debt has been reduced during the past year from £1,677 to £1,084. The expenditure for the year was £7,134 and the income £7,882. 1471 patients were treated in the wards at an average cost of £4 17s. The convalescent home which was established three years ago in connection with the hospital has taken charge of 130 patients, whose earlier discharge from the hospital enabled a larger number of acute cases to be treated.

### TAUNTON.

**Water Supply.**—A new storage reservoir, capable of holding 120,000,000 gallons, in connection with the Taunton water supply, was formerly opened by the Mayor on June 30th. The reservoir is situated at Laxhay, on the Blackdown Hills, eight miles from Taunton. It will have a water area of 17 acres when filled, with a depth of water of 57 feet. The cost of the undertaking has been about £26,000.

